# Study the Antinociceptive Effect of Intracerebroventricular Injection of Aqueous Extract of *Origanum Vulgare* Leaves in Rat: Possible Involvement of Opioid System

**Published:** 2013-10

**Authors:** Yasaman Pahlavan, Gholamreza Sepehri, Vahid Sheibani, Mohammadreza Afarinesh khaki, Morteza Gojazadeh, Bahare Pahlavan, Fereshteh Pahlavan

**Affiliations:** 1 Neuroscience Research Center and Department of Physiology, Faculty of Medicine, Kerman University of Medical Sciences, Kerman, Iran; 2 Department of Physiology, Faculty of Medicine, Shahid Beheshti University of Medical Sciences, Tehran, Iran; 3 Departments of Physiology, Faculty of Medicine, Tabriz University of Medical Sciences, Tabriz, Iran; 4 Faculty of Medicine, Tabriz University of Medical Sciences, Tabriz, Iran; 5 Faculty of Medicine, Ardabil Azad University of Medical Sciences, Ardabil, Iran

**Keywords:** Intracerebroventricular, *Origanum vulgare*, Pain, Rat, Tail-flick test

## Abstract

***Objective(s): ***The aim of study was to investigate the antinociceptive effect of intracerebroventricular (ICV) microinjection of *Origanum vulgare* (ORG) extract and possible involvement of opioid receptors.

***Materials and Methods: ***Cannula was inserted into left ventricle of male rats. Five days after surgery Tail Flick Latency (TFL) was measured after ICV microinjection of, ORG (1, 3 and 6 µg / rat). Effective dose of ORG was injected ICV in concomitant with morphine (2 mg/kg, IP), naloxone (2 mg / kg, IP) and saline (0.5 µl/rat) and TFL was recorded.

***Results: *** The co- administration of ORG extract with morphine showed a significant increase in TFL and naloxone, pretreatment significantly inhibited the antinociceptive activity of ORG and morphine.

***Conclusion: ***The aqueous extract of ORG possesses antinociceptive activities in the tail-flick test in a dose dependent manner. ORG - induced antinociception may have been mediated by opioid systems.

## Introduction

Medicinal plants and traditional herbs have gained importance in the last decade as therapeutic agents. With increasing demand in the field of herbal medicines and cosmetics, it has become necessary and pertinent to probe into the area of systematic knowledge about herbal drugs. *Oregano* is species of *Origanum* of the *mint* family and a native to Europe and Mediterranean region, Southern and Central Asia (1). There are over 40 species of *oregano*, but the essential oil produced from *Origanum vulgare* (ORG) is considered to be the most therapeutically beneficial. The oil is extracted from the dried flowering herb by steam distillation (2-4).

Hernandez-Hernandez *et al* (2009) reported the antioxidant effect of *oregano* leaves (ORG L.) and the antioxidant effect of the studied extracts depends, not only on the concentration of phenol compounds (rosmarinic acid, carnosol and carnosic acid), but also on the extraction method and solvent (5). Other investigators reported the antibacterial activity (6), antioxidant (5, 7), antimelanogenesis activities of protocatechuic acid from ORG (oregano), treatment of urolithiasis by preventing polyurea, crystalluria, oxaluria, raised serum urea and creatinine levels and crystal deposition in kidneys (2). Traditionally leaves of ORG is a medicinal plant used in Iranian folk medicine as a pain reliever, however, there is no documented data regarding its possible antinociceptive mechanism(s). So the present study sought to assess antinociceptive effect of ICV microinjection of ORG extract and possible involvement of opioid receptors in its antinociceptive mechanism(s) using tail flick test.

**Table 1:** The effects of ICV microinjection of *Origanum vulgare* aqueous extract on tail flick latency in rat

Groups	Tail flick lastency ( Sec)					
	Min 30	Min 45	Min 60	Min 75	Min 90	Min 120
Control	3.94+0.32	4.07+0.39	3.50+0.20	3.54+0.28	3.97+0.42	4.20+0.51
ORG ( 1mg/kg)	3.71_0.75	4.05+0.32	3.54+0.37	4.05+0.63	3.55+0.24	3.64+0.19
ORG ( 3mg/kg)	6.02+1.06	5.47+0.95	5.58+0.82 *	4.79+0.88	5.80+0.86 *	5.17+0.86
ORG ( 6mg/kg)	5.43+0.97	4.56+0.32	5.06+0.72	4.89+0.23	3.92+0.42	3.84+0.32

Antinociceptive effect of ORG was evaluated after ICV microinjection of 1, 3 and 6 µg / rat of aqueous extract of ORG and TFL was measured after 30 min for 120 min. Control group received the same volume of saline intraventricularlly . Data expressed as mean± SEM of 7 rats in each group

ICV= intracerebroventricular, ORG = Origanum vulgare, TFL= tail flick latency

*= *P*< 0.05 compared to control

## Material and Methods

***Animals***

All experiments were carried out on male Wistar rats, weighing 200-250 g, that were housed four per cage under a 12 hr light/dark cycle in a room with controlled temperature (22±1°C). Animals were fed with standard diet (Lipton Feed) and tap water.

Rats were divided randomly into six experimental groups, each comprising 7 animals. All of the procedures were in accordance with guidelines for caring and using of laboratory animals in Neuroscience Research Center of Kerman University of Medical Sciences and the European Communities Council Directive of 24 November 1986 (86/609/EEC).

The rats were anaesthetized by ketamine (80 mg / kg/IP) and xylazine (10 mg / kg/IP). Then a stainless steel, thin-walled guide cannula was inserted into left ventricle according to Paxinos and Watson characteristics using stereotaxic apparatus (AP = - 0.9 mm, DV = - 3.5 mm, Ml = - 1.2 mm)(8). The animals were allowed to recover from surgery for 5-7 days prior to initiation of experimental protocol.

***Antinociceptive test***

Antinociception was assessed by tail-flick test. Radiant heat was applied at 5-8 cm from the tip of the tail using a tail-flick apparatus (PANLAB 7160, Spain). Tail flick latency (TFL) was measured as the time of the beam exposure to the withdrawal time of the tail. The mean of three consecutive TFL was measured at 1-min intervals before drug or solvent administration (basal latency) and then similar TFL was measured at specific times after drug or solvent administration, (experimental latencies)(9). The intensity of radiant heat was adjusted to establish the basal latency of 3-5 sec. To avoid tissue damage, a cut-off time of 10 sec was set. Trials were automatically terminated if a response did not occur within 10 sec (9).

Forty-nine male Wistar rats (200-250 g) were divided into 7 groups (n = 7): control, ORG (1, 3 and 6 µg / rat), morphine+ ORG and naloxone + ORG and saline + ORG treated rats.

Antinociceptive effect of intracerebroventricular (ICV) microinjection of ORG was evaluated after 1, 3 and 6 µg / rat of aqueous extract of ORG. Control group received the same volume of saline intraventricularlly and TFL was measured after 30 min. To determine the possible involvement of opioid receptor in ORG antinociceptive effect, the most effective antinociceptive dose of ORG (3 µg / rat) was injected ICV with either morphine (2 mg / kg/ IP) or naloxone (2 mg / kg /IP) and saline (0.5 µl/rat) and TFL was measured by the same protocol. The aqueous extract of ORG was administered 20 min after drug or vehicle injection. The TFL of rats to thermal stimulation of the tail was recorded before, and 30, 45, 60, 75, 90 & 120 min after treatment by tail-flick test. All drugs and solutions were freshly prepared before each experiment.

***Preparation of ORG extracts***

Leaves of ORG were collected at spring in Yazd city. The plant was identified and confirmed by a botanist in the biology department of Shahid-Bahonar University (Kerman, Iran) as ORG. Leaves were dried in shade and powdered. Powder was refluxed with distilled warm water (below 50°C) by 1 / 100 ratio for 24 hr. The extract was filtered with Whatman No. 2 filter paper. The mixture was concentrated under reduced pressure at 40°C by a Rota evaporator. Evaporation gave a semi-solid mass yielded 8 % W/W. Stock solution of the extract was prepared by dissolving 5 g of extract in 100 ml of distilled water to prepare a 50 mg ml-1 concentration. Other concentrations were made from this stock solution by appropriate dilution with distilled water.

**Table 2:** The effects of injection of morphine or naloxan (IP)/ Origanum vulgare extract on tail flick latency in rat

Groups	Tail flick lastency ( Sec)					
	Min 30	Min 45	Min 60	Min 75	Min 90	Min 120
Control	2.86±.57	3.36± .43	3.11± .46	2.98± .38	2.77±.44	2.79± .65
(Saline+ORG)	4.25± .49*	3.75± .38*	3.63±.44	3.03± .27*	2.29±.212*	1.77±.410*
Morphine+ORG	4.63±.93	4.94± .79	4.05± .57	3.40±.31	2.57± .34	1.88±.401
Naloxan+ORG	2.74± .33	2.48 ±.36	2.34± .48	2.08± .37	1.45± .15	1.42± .20

Antinociceptive effect of ORG was evaluated after ICV microinjection of 1, 3 and 6 µg / rat of aqueous extract of ORG and TFL was measured after 30 min for 120 min. Control group received the same volume of saline intraventricularlly . Data expressed as mean± SEM of 7 rats in each group

ICV= intracerebroventricular, ORG = Origanum Vulgare**, ** TFL= tail flick latency

*= *P*< 0.05 compared to control

Repeated measurement test was used to determine significant differences between groups. One way analysis of variance followed by the multiple comparison test of Tukey - Kramer was used to determine significant differences within group. The data are expressed as Mean ± standard error of the mean (SEM). *P < *0.05 was considered statistically significant.

## Results

Antinociceptive effect of intracerebroventricular (ICV) microinjection of ORG was evaluated after 1, 3 and 6 µg / rat of aqueous extract of ORG. Control group received the same volume of saline intraventricularlly and TFL was measured after 30 min for 120 min. The result of this study showed that ICV microinjection of ORG extract resulted in significant and dose - dependent increase in the TFL in the tail-flick test and significant effect was observed at a dose of 3 µg / rat (*P< *0.001). The maximum TFL was observed at approximately 60 min(5.58 ± 0.82 in ORG vs 3.50 ± 0.20 in control group) and 90 min (5.80 ± 0.86 in ORG vs 3.97± 0.42 in control group) after 3 µg/rat ORG microinjection (*P < *0.001)(Table 1).

There was significant increase in the pain threshold or the TFL following the co - administration of ORG extract with morphine in the tail-flick test 30 min after drug treatment ( 4.63 ± 0.93) as compared to control (2.86 ± 0.57) and ORG (4.25± .49) treated rats (*P >*0.05) (Table 2). 

Naloxone, (2 mg / kg/ sc) pretreatment inhibited the antinociceptive activity of ICV microinjection of ORG (3 µg / rat) extract. TFL in naloxone treated rat was significantly decreased as compared to ORG(2.34±.48 in naloxone vs 3.63±.44 in ORG group) and morphine + ORG(2.34± .48 in naloxone vs4.05±.57 in morphine + ORG group ) 60 min post drug treatment(*P >*0.05) (Table 2).

## Discussion

The results of this study showed that ICV injection of ORG extract showed analgesic activity in a dose dependent manner and maximum antinociception was observed in 60 and 90 min after ICV injection and significant effect was observed at a dose of 3 µg / rat (*P< *0.001). The maximum TFL was observed at approximately 60 min and 90 min after 3 µg / rat ORG microinjection (*P < *0.001). Other studies have reported that some of ORG family plants have antinociceptive effects (10-12). Antinociception efficiency is related to time and dose of injection.

This result is in agreement with studies of other researchers (11, 12). Arzi *et al* (2009) demonstrated that administration on hydroalcoholic extract of ORG showed analgesic effect in formalin test (12). Mikaili *et al* (2010) showed that IP injections of ORG (1, 1.5 and 2 mg/ml) caused significant analgesic effect in acute pain model by tail flick test (11). In our study, the maximum TFL was observed at approximately 60 min and 90 min after 3 µg / rat ORG microinjection which is in complete agreement with previously reported results in mice (11). ORG is among the traditional Persian medicine which is used for the pharmacological treatment of headache (13). Also others reported that thymol, α-pinene and carvacrol composition of ORG showed high antibacterial activity (14).

The mechanism(s) of antinociceptive effect of ORG is not determined yet, however, it may be mediated through the chemical composition of the essential oils of ORG, mainly its carvacrol/thymol constituent (6). Others also reported the antinociceptive effect of carvacrol (CARV) (5-isopropyl-2-methylphenol) in several models of pain in mice (10).

Guimarães *et al* (2010) reported that CARV reduced the number of acetic acid-induced abdominal writhing significantly compared to the control group, without opioid participation in mice (*P< *0.001). In the formalin test, CARV also significantly inhibited both early (neurogenic pain) and late (inflammatory pain) phases of formalin-induced licking for the inflammatory phase. CARV also produced a significant inhibition of pain caused by capsaicin (*P< *0.001) and glutamate (*P< *0.01)(10). Similarly Melo *et al* (2012) showed that carvacrol produced significant inhibitions on nociception in the acetic acid-induced abdominal constriction, formalin and hotplate tests in mice, however, in the open-field and rotarod tests, carvacrol did not significantly impair the motor performance (15).

Antinociception in the tail-flick test is thought to primarily involve spinal mechanisms, thus, the present results primarily show the possible involvement of spinal mechanisms of ORG-induced antinociception, while antinociception induced by ORG oil or extracts may also to be mediated through supraspinal mechanisms (i.e. brain). Accordingly, it was of high priority and importance to test the analgesic effect of ORG oil or extracts by the method of hot plate late test, which may reveal central nature of antinociceptive activity of ORG extract which could be considered as the first limitation of our study. Also, study of analgesic activity of ORG extract following its intrathecal administration is recommended. 

Our results showed that co - administration of ORG extract with morphine caused a significant increase in TFL in the tail-flick test as compared to control and ORG treated rats (*P<*0.05) and naloxone pretreatment revealed a significant reduction in the TFL of ICV microinjection of ORG extract which shows that a part of ORG extract antinociception could be mediated through opioid participation. Although there is no documented data on involvement of opioid receptors in CARV antinociception mechanism in acute pain, however, others showed that CARV (as the main ORG chemical composition) antinociception in acetic acid-induced abdominal writhing is not mediated though opioid receptors (10), so it is proposed that other chemical composition of ORG such as thymol/alpha-terpineol; linalyl acetate and linalool, carnosic acid and carnosol, germacrene D, beta-ocimene and sabinene or beta-caryophyllene and unidentified active compounds may be involved in its antinociception effect through opioid system. Also its antinociception could be mediated through the modulation or inhibition of the pain caused by capsaicin and /glutamate (1, 5, 6, 10).

Although other researchers reported that carvacrol, the main constituent of ORG extract, did not significantly impair the motor performance in the open-field and rotarod tests, however, the responses observed following administration of ORG may be due, at least in part, to some changes in behavior of the mice. So it would be better to determine whether or not the crude ORG extract alter the mice motor coordination and the spontaneous motility and this is the second limitation of our study.

In other reported that ORG extracts possess the highest antioxidant capacity, possibly due to the presence of high concentrations of carnosic acid and carnosol, germacrene D, beta-ocimene and sabinene or beta-caryophyllene and unidentified active compounds (1, 5). Still others have reported that Thymol, α-pinene and carvacrol composition of ORG showed higher antibacterial activity than streptomycin(14). 

## Conclusion

The results of our study showed that the aqueous extract of ORG possesses antinociceptive activities in the tail-fli**c**k test in a dose dependent manner. Also our results showed that naloxone pretreatment revealed a significant reduction in the TFL of ICV microinjection of ORG extract which shows that a part of ORG extract antinociception could be mediated through opioid participation. 
